# Early Intervention in Post-operative Infectious Spondylodiscitis: Outcome of Aggressive Transforaminal Lumbar Interbody Fusion

**DOI:** 10.5704/MOJ.2411.003

**Published:** 2024-11

**Authors:** A Krishnan, BR Dave, D Degulmadi, S Mayi, R Rai, P Bang, M Dave, V Chauhan, S Bali, P Charde, A Anil, P Krishnan

**Affiliations:** 1 Department of Spine Surgery, Stavya Spine Hospital and Research Institute, Ahmedabad, India; 2 Department of Radiodiagnosis, Stavya Spine Hospital and Research Institute, Ahmedabad, India

**Keywords:** post-operative, spondylodiscitis, lumbar, discectomy, TLIF

## Abstract

**Introduction:**

Conservative and surgical approach timeline in post-operative spondylodiscitis (POS) following lumbar disc herniation (LDH) surgery is ill defined, and patients have a protracted recovery phase with social, psychological, and financial implications.

**Material and Methods:**

Retrospective analysis of patients operated by transforaminal lumbar interbody fusion (TLIF) in POS was done. Confirmed clinico-radiological diagnosed POS cases, not responding within three to four weeks were included. Normalisation of CRP and radiological stable reconstruction was assessed for objective clearance of POS and bony union.

**Results:**

Ninety-five patients were included in the study with minimum follow-up period of two years. The mean age was 51.63±13.63 years. There were organisms cultured in 55 patients (57.89%). The ODI improvement of the patients was noted to improve from 88.71±5.3 to 20.80±9.7 (8 weeks) and was incremental at 2 years follow-up (10.12±6.41) and maintained further at final follow-up at 9±4.3. Bony union achieved in all with stable reconstruction. The resumption of activities of daily living (ADL) was quick (15.90±8.20 days) and job (3.67±1.31 months) was achieved in all the patients. In poor outcomes, two patients didn’t respond, and one patient died due to uncontrolled infection.

**Conclusion:**

Early diagnosis and intervention is the key to effective management of POS. Utilisation of aggressive TLIF yields faster ADL resumption.

## Introduction

POS following LDH discectomy is rare but serious complication with incidence reported from 0.21-3.6%^1-4^. In POS, Staphylococcus aureus is the leading causative organism followed by gram negative (Gm-ve) bacteria^4-7^. Patients of POS are characterised by severe back pain, leg pain, neurological affection and varied constitutional symptoms. The early and accurate diagnosis depends on a combination of clinical, laboratory, and imaging findings^5,8,9^. Surgeons conservatively tend to initiate a cocktail of antibiotics, prolonged bed rest and bracing from 4 weeks to 24 weeks^8,10^. Surgical fusion is resorted in individuals with non-improving sequel^11,12^. POS literature has abundant reports of conservative treatment, endoscopic approach, surgical debridement only and aggressive fusion surgeries^13-16^. The reports of good outcome of TLIF (Transforaminal Lumbar Interbody Fusion) surgeries in POS are favourably reported as well^16-18^.

There seems to be no consensus on the various surgical options and how long to do conservative treatment. Long term disability, problems of non-ambulation including psycho-economic effects put the surgeon and the recently operated patient into an unknown unpredictable path of recovery.

The aim of our study is to retrospectively find the outcomes of spondylodiscitis patients treated by early aggressive transforaminal interbody fusion. Additionally, to search literature to find if any consensus on the timeline which exists for the treatment of POS.

## Materials and Methods

Retrospective analysis of POS patients with minimum follows up of two years, who were operated by TLIF between April 2004 to May 2020 was done. The data was collected from our hospital records. The patients were labelled as a case of POS if, in the post-discectomy period of OD/MLD/TAD (open discectomy/microlumbar discectomy/Tube assisted discectomy), infective features developed. This meant, in the post-operative period with or without an initial recovery of few days, the patient had within the ensuing eight weeks developed clinical features of severe back pain with constitutional symptoms of fever and significant effects on activities of daily living (ADL). Classic radiological features on radiograph and MRI included one or more of the disc space narrowing, instability, vertebral destruction, signal changes in disc space, adjacent vertebral body oedema, end plates erosion, and/or para-vertebral soft tissues/pus. Surgery was offered to all those patients who presented to us and who did not respond to antibiotics for three to four weeks. All demographic data including age, sex, duration from index surgery on presentation to second surgery, significant disability days before index LDH surgery, interval between index and final TLIF surgery was recorded. Presence of other symptoms/ signs of radiculopathy and deficits, with any co-morbidities, level of surgery, whether they were operated at our own hospital or presented from other hospital and biopsy (wound culture-sensitivity (CS)/ Biopsy CS / Blood CS) were noted. All the patients were evaluated with radiographs and MRI. Visual Analog Scale (VAS) score for lower Back Pain (LBP) and leg pain was taken pre-operatively. Evaluation of neurologic and functional outcomes was done using the validated measures of Oswestry Disability Index (ODI). MRC scale was used for muscle power grading in 5 grades.

All patients were operated under general anaesthesia in prone position. A midline exposure of with single segment spinal fusion with pedicle screws and either bone graft or bone graft with cage was performed in all the cases. Complete laminectomy and one sided facetectomy done. After neural decompression, intervertebral disc was removed trans-foraminally, and end plates were prepared. The tissue from the disc space was bottled to send for culture (Bacterial/Fungal/Anaerobic/Tubercular organisms) and histo-pathological examination. Locally harvested posterior element graft was morselised and used for interbody fusion. If inadequate posterior elements were present, then iliac crest graft was used. In case of dry disease or not very purulent disc or without severe septic features, titanium cage was preferred for anterior reconstruction with bone graft (Cage[A] Group). Otherwise, only bone graft was put, and tri-cortical facet bone graft was positioned optimally (Bone graft[B] Group). Closure was done under a drain.

All patients were mobilised on the second post-operative day as tolerated. Appropriate antibiotic treatment was given as per the culture sensitivity report for duration of eight weeks. Calcium and Vitamin D3 were given to all as per recommended daily allowances and bisphosphonates were added after four weeks (above 50 years female and 60 years male) and continued for three years. Subcutaneous Teriperatide 20mcg injection daily was given in osteoporotic patients who were noted to have suboptimal screw hold per operatively and they were put on bisphosphonates afterwards sequentially. These patients were rested and braced with lumbosacral corset for three months. The response to antibiotic therapy was judged by the declining values of inflammatory markers (ESR/CRP) and with an improving mobility/decreasing pain. OR (operating room time from incision to closure in minutes) was noted. ODI score (preoperative to index surgery, pre-operative at TLIF surgery, eight weeks, two years and at final follow-up) was used to quantify clinical outcome. A patient satisfaction index was used as a self-assessment tool to determine the overall satisfaction outcome^19^. An infection was considered cured with improved clinical features, settled inflammatory markers (CRP) and stable radiograph. than compared with age, gender, and osteoporosis. Stability was assessed with screws/ cage position. The final radiological outcome was accepted stable and fused if no peri-screw loosening/ broken implant was present, and the stabilised segment showed static cage with appreciable inter- corporeal bone formation. The days taken for resumption of basic ADL within house activities (in days) after TLIF and resumption of previous activity/job (in months) were analysed. Complications, if any were noted and managed accordingly. Failure to respond to treatment was considered as complete failures.

Patient demographics and characteristic categorical variables were analysed, and the mean+SD (standard deviation) (minimum-maximum) for all applicable variables were calculated. Each category was compared by using appropriate statistical tools such as the Pearson correlations, unpaired Student t-test, and paired t-tests. The level of significance was considered p<0.05 for all tests. When the data wasn’t evenly distributed, the comparisons were made using Mann-Whitney Test. The data were checked for normality using Shapiro Wilk test and histograms. The data were compared across the groups of interest using two sample t-test adjusted for multiple comparisons using Bonferroni’s correction, if needed. In case of deviance from normality, non-parametric equivalents of Mann-Whitney test were used. The patients who were treatment failures or did not remain with us for further outcome were excluded from outcome calculations but mentioned in complications. Statistical analysis was performed with IBM SPSS software ver. 20.0 [IBM Corp, Armonk, NY, USA].

## Results

Ninety-five patients were included in the study and the demographic profile of the operated patients is tabulated (Table I). All patients were significantly disabled and had classic radiological features of POS. Before presenting to us, the number of patients investigated with a wound CS were 3 (4.2%) and with transpedicular / trans discal biopsy (TB) CS were 7 (14.08%) before the 72nd number of patients in the consecutive series (till 2018). Whereas the number of patients investigated with wound CS was 1 (4.1%), with Blood CS was 1 (4.1%) and with transpedicular/ trans discal biopsy CS were 9 (37.5%) afterwards amongst the 24 remaining patients after 2018. The fever at presentation was associated with culture growth (p=0.00069) in 42% (n=55) patients as compared to 10% (n=45) with no culture growth. The patients with no grown culture had CRP of 25.75±18.08, whereas the patients with grown culture had CRP of 53.19±31.33 (P<0.001). There were 40 (42.10%) patients with no organism cultured and 55 (57.89%) with cultured organism. The culture grown patients had ESR of 65.25±21.00, as compared to patients with no culture growth 69.18±20.66 (p=0.307). CRP in all responsive patients reached to normal level quantitively within three months. Though, ESR of the patients decreased but did not come to normal range in 41 patients. There were 23 patients with gram negative (Gm-ve) and 32 with gram positive (Gm+ve) organism culture.

**Table I:** Demographic features of all patients of POS (n=95).

Age (years)	51.63 ± 13.63 (22-82)
Sex (M: F)	47:48
Disability before Index surgery (Days)	31.9±17.54 (14-96)
High fever after discectomy	(n=95) 100%
Fever at presentation to us after conservative treatment	Present: 28.42% (n=27), Absent: 71.57% (n=68)
ODI Pre-operative to Index surgery	80.68±12.06 (55.56-95.56)
ESR at presentation	67.51±23.48 (20-76)
CRP at presentation	65.77±23.27 (18-133)
Pre-operative to TLIF	
VAS Score: Low Back Pain	9.23±1.52 (8-10)
VAS Score: Leg Pain	1.15±1.37 (0-5)
ODI Pre-operative to TLIF	89.14±5.39 (82.22-100)
Pre TLIF-Biopsy/ Culture reports	No Biopsy / tissue specimen report done in 78.94% (n=71),
	Transpedicular/ Discal Biopsy =20% (n=19),
	Wound Culture=4.21% (n=4)
	Blood Culture=1.05% (n=1)
Index discectomy Surgery	At Author’s Institute=5.26%(n=5),
	Other institute operated cases=94.73% (n=90)
Operated twice or more Comorbidities	6.31% (n=6) Ischemic heart disease 3.15 %(n=3), Thalassemia minor 1.05% (n=1), Diabetes mellitus 16.84% (n=16), Hypertension 23.15%(n=22), Renal Transplant 1.05% (n=1), Asthma 2.10% (n=2), Hypothyroid 8.42% (n=8), Skin 4.21% (n=4), Polyarthritis 10.52% (n=10), Smoking 9.47% (n=9), Tobacco 15.78% (n=15)

Abbreviations - ODI: Oswestry disability index, TLIF: transforaminal lumbar interbody fusion, VAS: visual analogue score

**Fig. 1:**
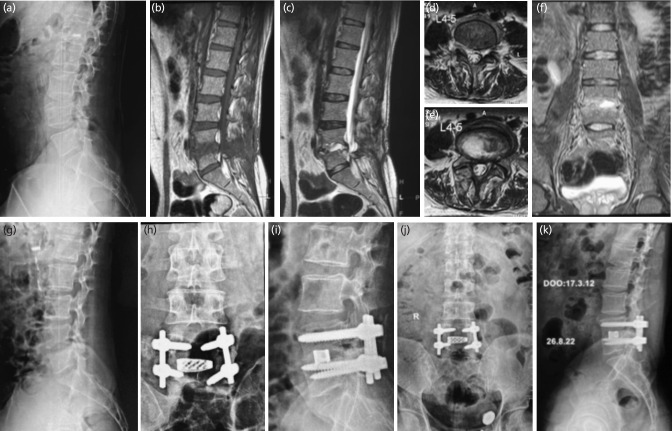
Male 22 years, operated micro lumbar discectomy for lumbar disc herniation L4-5. (a) Lateral radiograph before index surgery. (b-f) MRI T1-T2 sagittal, T2 axial and coronal sequences showing intra-discal abscess, left para-discal foraminal abscess, with end-plate destruction. (g) Lateral radiograph showing evident disc space narrowing and irregular end plate. (h, i) Antero-posterior and lateral radiograph showing transforaminal lumbar inter-body fusion executed with bone graft and cage with pedicle screws. (j, k) 95 months final follow-up antero-posterior and lateral radiographs showing stable united reconstruction.

**Fig. 2:**
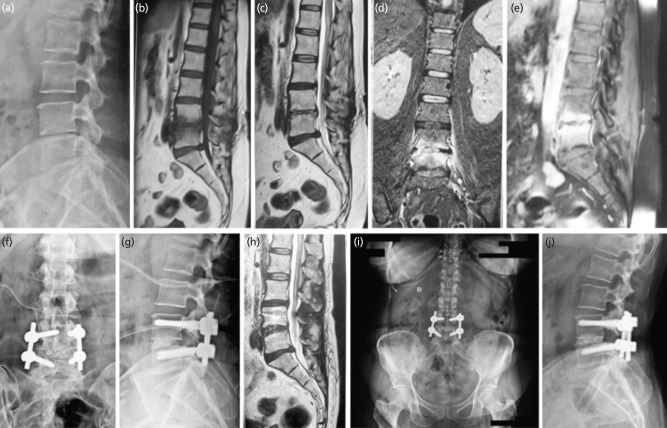
Female 48 years, Operated micro lumbar discectomy for Lumbar Disc herniation L4-5. (a) Lateral radiograph before TLIF surgery. Irregularity of endplate and disc space reduction noted. (b-d) MRI T1-T2 sagittal, coronal sequence showing intra-discal abscess, vertebral body edema, with end-plate destruction. (e) Contrast MRI showing soft tissue, body and disc enhancement. (f, g) Antero-posterior and lateral radiograph showing transforaminal lumbar inter body fusion executed with only interbody bone graft and pedicle screws. (h) MRI T2 sagittal at 3 months follow-up showing no active signs of infection. (i, j) 80 months final follow-up antero-posterior and lateral radiographs showing stable union and reconstruction.

The organism cultured and their category is tabulated (Table II). Surgical variables are tabulated in (Table III). Ninety-two patients had follow-up beyond two years. Three patients were failures in our treatment. Out of all the patients included in our study, only five patients had their initial surgery done at our institute. The rest of the patients had their surgery done at another institute and came to us for further management. The ODI improvement was statistically significant. No significant difference was observed between the A Group and B group for ODI improvement (Table IV) (Fig. 1, 2). No significant difference was observed in the final ODI score of the patients with osteoporosis (n=34) (10.69+4.57) and without (8.37+4.27) osteoporosis (p=0.137). The ODI after 8 weeks was not different (p=0.757) between patients with culture grown or not grown patients. No difference was there in DHL between A group and B group (p=0.732). The DHL or implant loosening was not related to age, sex, and culture growth but only related with osteoporosis (p=0.015). The DHL was compared across age, gender, osteoporosis and the prevalence of osteoporosis increased the odds of having a DHL more than 2mm by 4.4 times (p=0.015). No difference for resumption basic ADL or work resumption across gender were noted (p=0.106 and 0.438, respectively). The older patients (>60 years) took statistically similar time to resume ADL (17.95±4.57 days) as compared to younger patients (15.94±5.06 days) (p=0.100). They also took similar time to resume work (3.71±1.19 months) in comparison to younger patients (3.59±1.05 months) (p=0.775). All outcome variables are tabulated in (Table V).

**Table II:** Positive cultured organisms and their frequency.

Organism/s	Number of patients (n=55)
MSSA: Methicillin susceptible staphylococcus aureus	10
MRSA: Methicillin resistant staphylococcus aureus	11
Staph E: Staphylococcus epidermidis	5
E.Coli: Escherichia coli	13
Enterococcus faecium	1
Pseudomonas Aeruginosa	8
Aspergillus	1
Candida Albicans	1
Klebsiella	2
MTB Mycobacterium tuberculosis	3
Multiple organisms (MSSA+ E.coli; MTB+E.coli: Enterococcus faecium+E.coli)	3*

*: Patients with more than one organisms in culture report, they are individually also charted above.

**Table III:** Surgical variables of patients.

1st to 2nd surgery interval (Days)	36.58±23.69 (11-94)
Operated spinal level	L2-3 7.36% (n=7), L3-4 14.73% (n=14),
	L4-5 57.89% (n=55), L5-S1 20% (n=19)
Surgical method (TLIF with bone graft and cage:	
Group A, TLIF with bone graft only: Group B) Group B 33.68% (n=32)	Group A 66.31%(n=63)
Operation time (Minutes)	87.13±18.72 (63-130)
Estimated Blood Loss (ml)	201±61.44 (110-400)
Screw strength low/ osteoporosis	35.78% (n=34)
Culture organism growth	Gm +ve (n=32), Gm-ve (n=20), Both (n=3), No growth(n=40)
Histopathology	Acute chronic discitis=48, Discitis=44, Granulomatous=3

Abbreviations - TLIF: Transforaminal Lumbar Interbody Fusion, L: Lumbar, S: Sacral

**Table IV:** Surgical variables of patients.

	Pre-TLIF ODI	8 weeks ODI	2 years ODI	Final follow-up ODI
Group A	88.71±5.3.	20.80 ±9.7;	9.62 ±5.7;	9 ±4.3
Group B	90.19 ±5.88.	17.71 ±8.9.	11.11 ± 7.7;	9.53±4.8

Notes - TLIF: Transforaminal Lumbar Interbody Fusion. Seven patients were lost to current follow-up, but their minimum two years follow-up and later follow-up were available. Thus, they were included in calculation of ODI outcome.

**Table V:** Surgical outcome variables.

8 weeks ODI after TLIF	19.75±9.51 (6.67-33.33)
Follow-up ODI at 2 years	10.12±6.41(2.22-35.56)
Latest follow-up ODI	9.17±4.42 (2.22-24.44)
Follow-up (months)	46.90±28.44 (1-150)
Patient satisfaction index	1.25±0.43 (1-2)
Resumption of basic activities of daily living (Days)	15.90±8.20 (6-30)
Resumption of Job/ previous activities (months)	3.67±1.31(2-6.5)
Radiological Fusion Yes / No	Yes 100% (n=92)
Disc height loss at 2 years (mm)	<2mm (n=76, 80%),2-4mm (n=14, 14.73%), 4mm (n=2, 2.10%)
Cage Implant Position	Fully Stable 90.52% (n=86), Back out but stable 6.31% (n=6)

Complications were in the form of wound dehiscence in four patients, five patients had sinus at the operative scar which healed spontaneously, four patients had urinary tract infection, one patient died due to septicaemia and uncontrolled infection. In two patients where infection was not controlled, and they did not consult us and lost to follow up (E. Coli). Thirteen patients had poor operative screw hold after the surgery due to disease osteoporosis and 21 patients were known osteoporosis on documented DEXA scan (Dual energy X-ray absorptiometry).

## Discussion

POS was first reported by Turnbull in 1953^20^. Several patient-related risk factors play role in pathogenesis of infection that may be modifiable or non-modifiable. Non-modifiable factors include advanced age, immunosuppression, urgent surgical need and diabetes mellitus. While, obesity, smoking, indwelling catheters, malnutrition, administration of antibiotic and prolonged hospitalisation are all modifiable factors^5,20,21^. Our patients also had comorbid factors in 36.84% (n=35) patients. Although, the spectrum of clinical presentation of POS is variable, but excessive pain and refusal to perform any physical movement should raise the index of suspicion^1,4,6^. Post-operative fever cannot always be due to wound infection. Only sustained fever (above two weeks) should be suspected to be infection^22^. Fever was present in all our patients in the first three weeks after surgery. But 71.57% (n=68) patients responded and had no fever when they presented to us. Invariably all patients were treated with empirical antibiotics. The duration of the post-operative pain relief may vary from a few days to 10 weeks after index LDH surgery^1,4,8,23,24^. The severe back pain at presentation was present in all our patients and was above an average of 9.23 VAS LBP score.

The radiographic finding which may first appear is loss of intervertebral height with erosion plates^1,4^. Though plain radiographs fail to diagnose spondylodiscitis until two to eight weeks after onset of symptoms, a CT scan does help in early diagnosis^25^. MRI is the choice of radiographic modality for diagnosing POS^5^. Operated level shows more or less changes due to surgery itself and triggering post-operative inflammatory response^26^. In early disease with high clinical suspicion of infection FDG PET/CT might be preferred as first line of imaging over MRI for diagnosing POS^27^. All our patients had typical features of POS on MRI. The CRP level elevation is normal after any spinal surgery which peaks for two to three days and normalise within one to two weeks, (CRP value of <2.5Ug/ml)^28^. Persistently elevated ESR and CRP values in a highly suspected case with typical changes on MRI strongly suggested the diagnosis^5^. Though ESR has low specificity, it is used to diagnose and follow-up cases including infection, inflammatory disease, malignant tumour, and trauma. More importantly it’s the normalisation of ESR and CRP is to be monitored^14^. Novel tests like serum amyloid A, presepsin, procalcitpnin, 18F-fluorodeoxyglucose (FDG) positron emission tomography etc. can be used in limited set ups and soon will find wider applications after validation for post-operative spinal infection^23^.

In our study all patients had elevated markers CRP and ESR. We sequentially monitored CRP/ESR and the results of CRP which became normal in all responding patients (n=92) by 12 weeks. Though, ESR reduced but not to normal values in 41 patients. ESR can be non-specific in Indian set up and it is not uncommon to get a raised ESR^29^. Twenty-seven patients had fever at presentation to us and their CRP was significantly higher than the patients who did not present with fever (p<0.001). Culture growing patients in our series were having a higher value may be an indicator that active infection was still on, and the previous empirical therapy was not effective. The leading cause in most literature of spondylodiscitis including POS is Staphylococcus Aureus (60%) and gram-negative organisms^4,6,7^. In recent years, immunocompromised states and the use of broad-spectrum antibiotics have led to increase in the infection rate with unusual organisms including fungi and Mycobacterium Tuberculosis (MTB) species^7^. The organism most common cultured in our study was Staphylococcus Aureus (n=10). Most of the authors consider that the main cause of these infections is an inoculation during surgery and less frequently a hematogenous contamination^1,5,8,25^.

Only three cases of POS due to MTB are reported in literature^30-32^. In our study the MTB was noted in the culture of three cases. All these cases were operated at the rural centres for LDH. These surgeries were carried out within 17+17.34 (11-17 days) of presentation without probably adequate conservative trial. MTB is endemic in India^33^. The author suspects that these three POS cases might actually be primary spondylodiscitis which were not diagnosed at the rural centre before the initial surgery. It is the author’s recommendation that for patients presenting with disproportionate severe pain or surgery for LDH within three weeks, or where an epidural block has been given already, per-operative disc sampling for culture/ histopathology primarily may be of immense value when blood reports/MRI don’t raise any suspicion.

**Fig. 3:**
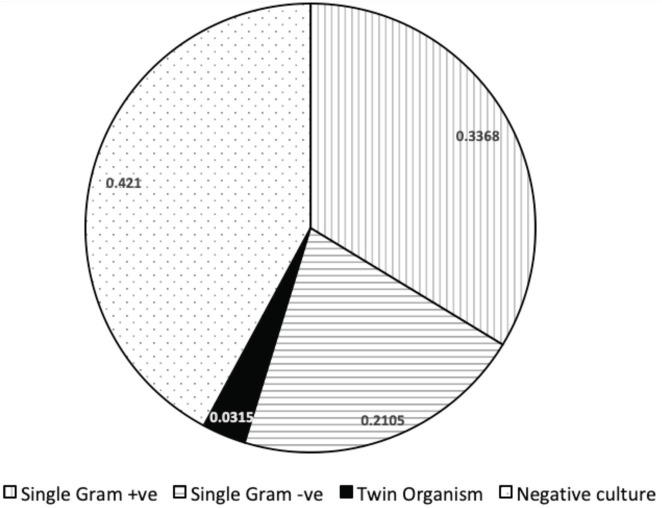
Pie Chart showing details of culture positive and negative patients.

In our study 55 patients had a positive culture report while 40 cases didn’t have one. Gm +ve organisms (n=32) were more in our series than Gm -ve organism (n=20) (Fig. 3). The culture not grown patients may be considered as aseptic discitis as well^34^. But our all cases (n=95) we had fever postoperative which lasted for variable days with severe back pain. Aseptic discitis is a less reported concept that has been postulated to be precipitated due to traumatisation of the disc and vascular compromise of the surgery^12^. A statistically significant 41% percentage of patients presenting with fever to us had yielded culture, as compared to only 10% who had fever, but culture did not grow. This could mean two possibilities. Firstly, the culture non growing patients were responsive to the previous empirical conservative treatment, but they had significant disability due to mechanical dysfunction of the affected spinal segment. Second possibility is that the culture non growing patients had aseptic discitis. In all probability it was the mechanical dysfunction of a spinal segment. The rate of biopsy pre-operatively (34%) was higher after 72nd number of the patient in the year 2018 in our series. Though in our own operated patients presenting with POS, biopsy was always done, and specific therapy was initiated. This shows that there was more standardisation in obtaining a sample before starting specific therapy in POS patients by practitioners in our region after 2018. The management of discitis is a challenge and matter of controversy and there is no universally accepted treatment protocol. Long periods of strict bed rest up to several months in conservative approach might lead to dramatic medical and psychological consequences. Additionally, major complications (e.g., colitis, renal failure, allergic reactions) as side effects of long-term antibiotic, non-specific therapy are also reported^35^. Surgical options for the patients of POS can be varied from endoscopic debridement to fusion. PTELD (Percutaneous transforaminal endoscopic lumbar discectomy) and irrigation can bring immediate pain relief and better outcomes for the patients, especially with co-morbidities in POS^36^. Though, it also does not describe an objective or subjective criteria till which time it can work. It has been previously published by the authors that transforaminal endoscopy can work in very early cases, but not if mechanical symptoms have already established and there is segmental dysfunction^37^. As discussed in the above section of aseptic discitis, mechanical dysfunction is in all probability the point of no return in the conservative care of POS. Once ensued, patients’ disability may last well over few months before the natural history of spondylodiscitis/POS can heal spontaneously^38^.

A timeline for fusion intervention cannot be inferred from the available literature, and there is a delay in aggressive management in POS in most of the series. In our study we did transforaminal lumbar interbody fusion (TLIF) in all patients. TLIF achieves a single-stage fusion through only a posterior approach. Early surgical intervention in the form of TLIF helped us to make the path more predictable at each step like from isolation of organism to early mobilisation. In our study, post-operative ODI when correlated with the method used for TLIF, there was no significant statistical difference between the patients operated with cage and bone graft as compared to those with bone graft only. The use of TLIF technique seems a good pathway to achieve complete debridement, access the disc space, bypass the scared zone, remove avascular disc, and simultaneously achieve solid circumferential fusion, avoiding the more complicated/unfamiliar anterior approach^15,17,39-42^. The mean operative time, mean blood loss, fewer complication rates in our series of 92 patients followed-up for a long term proves TLIF to be a very reasonable approach for management of POS. MIS-OLIF (minimal invasive spine surgery oblique lumbar interbody fusion) and ALIF (anterior lumbar interbody fusion) for Spondylodiscitis are also reported methods with good outcomes^43,44^. There is a report of achieving fusion with sampling and segmental stabilisation only^8^. Knowledge of osteoporosis in patients with pyogenic spondylodiscitis prior to surgery is very important as it might alter the surgical strategy (such as using multiple points of fixation, varied fixation equipment, and modified screw design/trajectories^43^. There is limited data reporting the coincidence of pyogenic spondylodiscitis and osteoporosis^27,44^. In our series the patients with osteoporosis (n=34), aggressive mobilisation was avoided. They were braced and were given teriparatide. They all responded well and in comparison, with the other patients did not show any outcome difference (p=0.137). Resumption of basics ADL (except in osteoporosis) and routine job / activity resumption was statistically unrelated to age, sex, osteoporotic, culture positivity or different organisms in our series. Three patients had uncontrolled infection, one died and two of poor outcome changed doctor. All these three patients had E. coli infection. The main difference in our study and those reported in literature is early aggressive surgery. None of the studies mention any optimum duration of conservative line of treatment. Although, many of those studies have concluded that conservative is the best line of approach, we noted that in all these studies up to 12% to 35% patients have finally ended up in surgery even after taking antibiotics for longer duration. None of these studies also mention about the resumption of basic ADL or job timeline^9, 45-48^. In other series up to 50% of patients were able to return to their normal routine and others had to stop working or take a lighter job and were vocational handicap^49-51^. Most patients with disc space infection progress to spontaneous interbody fusion over the period of 6 to 12 months^8^. But it can be very well inferred from all literature that once mechanical dysfunction sets in, then a prolonged time will take for self-stabilisation. The conservative treatment regime should not be attempted more than three to four weeks and delaying the ambulation and productivity could have long lasting implication to the patient in the form of burden of cost, disability, and psychological dysfunction.

Although, result bias has been avoided by complete assessment and analysis, there are limitations to the current study. This study was not a prospective study and no comparative study was done. Union was assessed by subjectively on conventional radiographs. CT scan would have been ideal to assess union. In none of the operated patients Teriperatide was given for full 24 months as recommended for osteoporosis. This was not done due to price consideration choice by the patient and non-compliance. But, with disease osteoporosis and implant stability in doubt, this approach was taken, and it worked out very apt as proven by the outcome in our study. Still, our series gives enough information to suggest validity for aggressive management of patients of POS not responding. Although a number of case series have been previously discussed, our series is the largest till date reported.

## Conclusion

Early diagnosis and intervention are the key to effective management for cases of post-operative spondylodiscitis. Utilisation of TLIF seems effective for post-operative recovery. Aggressive management yields faster resumption to ADL. Lesser invasive endoscopic debridement/open debridement alone or biopsy followed by conservative treatment should be compared with aggressive operative fusion in future studies to define their roles in management of early POS.
